# Body mass index (BMI) trajectories and risk of colorectal cancer in the PLCO cohort

**DOI:** 10.1038/s41416-018-0121-y

**Published:** 2018-06-06

**Authors:** Rui Zheng, Mulong Du, Baoguo Zhang, Junyi Xin, Haiyan Chu, Min Ni, Zhengdong Zhang, Dongying Gu, Meilin Wang

**Affiliations:** 1 Department of Environmental Genomics, Jiangsu Key Laboratory of Cancer Biomarkers, Prevention and Treatment, Collaborative Innovation Center for Cancer Personalized Medicine, Nanjing Medical University, Nanjing, China; 2 Department of Genetic Toxicology, The Key Laboratory of Modern Toxicology of Ministry of Education, School of Public Health, Nanjing Medical University, Nanjing, China; 3 Department of Biostatistics, School of Public Health, Nanjing Medical University, Nanjing, China; 4 Department of Oncology, Nanjing First Hospital, Nanjing Medical University, Nanjing, China; 5 Department of Colorectal Surgery, The Third Affiliated Hospital of Nanjing University of Chinese Medicine, Nanjing, China

**Keywords:** Cancer, Risk factors

## Abstract

Obesity is correlated with increased colorectal cancer (CRC) risk, but few studies have investigated lifetime body mass index (BMI) metrics and CRC risk. In a cohort of 139 229 subjects in the Prostate, Lung, Colorectal, and Ovarian (PLCO) Cancer Screening Trial, we analysed the effects of life-course BMI trajectories on CRC risk. At 13 years of follow-up, 2031 subjects developed CRC. Compared with subjects who were never overweight/obese, subjects who first exceeded the threshold of 25 kg m^−2^ at age 20 had a higher CRC risk (HR = 1.28, 95% confidence interval (CI) = 1.11–1.48). A body weight gain of ≥15 kg between 20 and 50 years of age (HR = 1.34, 95% CI = 1.18–1.52) and baseline (HR = 1.24, 95% CI = 1.08–1.43) was significantly associated with increased CRC risk. BMI trajectory analyses revealed that the CRC risk increased gradually over the three BMI trajectories (HR = 1.11–1.27, *P*_trend_ = 0.005) compared with subjects who maintained a normal BMI. Being overweight/obese at the onset of adulthood and BMI trajectories over the lifespan that result in obesity lead to an increased CRC risk.

## Introduction

Colorectal cancer (CRC) contributes to the global burden of cancer incidence, and despite declining rates of CRC in some developed countries, CRC incidence remains high.^1^ As revealed by epidemiological evidence, excess body fat (i.e., being overweight/obese) is a potentially modifiable lifestyle factor related to CRC.^2^ There are several methods to evaluate excess body fat, such as the body mass index (BMI), waist circumference, and waist-to-hip ratio, among others, although BMI is traditionally and widely used as a measurement for easy operation in large epidemiological studies.^3^ Previous studies have revealed that individuals with a higher BMI tend to have a higher CRC risk,^4,5^ but the relationships among changes to BMI trajectory, cumulative BMI measures during adulthood, and CRC incidence have not been investigated.

In this study, we explored the association of CRC risk with age-specific BMI, average BMI, body weight changes, and life-course BMI trajectories in a large prospective cohort study (the Prostate, Lung, Colorectal, and Ovarian (PLCO) Cancer Screening Trial).

## Materials and methods

### Study population

The PLCO study is a population-based cohort study that aims to evaluate the accuracy and reliability of screening methods for prostate, lung, colorectal, and ovarian cancer.^6^ This study was based on 70 541 subjects in the intervention arm and 68 688 in the control arm. The subjects completed a baseline questionnaire consisting of questions on demographic characteristics and CRC characteristics (Supplementary Materials). The usage of the CRC database in the PLCO study was authorised by the ethics committees of the data providers and Nanjing Medical University.

### Ascertainment of CRC

At study entry, subjects were not diagnosed with CRC. CRC cases were ascertained by self-reported annual questionnaires and linkage to the National Death Index (for completeness) and were histologically confirmed via medical record reviews (Supplementary Materials).

### Assessment of BMI

BMI was ascertained from self-reported questionnaires completed by the participants in the PLCO study.^7^ We divided the BMI values (kg m^−2^) at the three analysed age points into four categories according to the World Health Organization criteria: underweight (<18.5 kg m^−2^); normal (18.5 to 24.9 kg m^−2^, reference); overweight (25 to 29.9 kg m^−2^); and obese (>30 kg m^−2^). Weight changes during adulthood were classified as loss (≤−2 kg), stable (>−2 to <5 kg, reference), gain (≥5 to <15 kg), and notable gain (≥15 kg)^8^ (Supplementary Materials).

### Statistical analysis

Hazard ratios (HRs) and 95% confidence intervals (CIs) for CRC risk were estimated using Cox proportional hazard regression models to evaluate the relationships of age-specific BMI, average BMI, and weight change with CRC risk. To identify subjects with similar patterns of BMI development through the ages of 20 and 50 years and baseline, BMI trajectories based on the latent class growth model were generated with PROC TRAJ (SAS Institute, Inc., Cary, NC, USA).^9^ As a result, four trajectory categories (stable normal BMI, normal BMI to overweight, normal BMI to obese, and overweight to obese) were ultimately obtained.^10^ All the *P* values were two-sided, and the results were considered significant at *P* < 0.05. The analyses were performed using SAS version 9.4 (SAS Institute, Inc.) (Supplementary Materials).

## Results

### Subjects

This study tracked a total of 139 229 subjects and included 2031 cases. The age at trial entry was 62.6 ± 5.3 years, and the BMIs at the ages of 20 and 50 years and at baseline were 22.1 ± 3.0, 25.9 ± 4.2, and 27.3 ± 4.7 kg m^−2^, respectively (Supplementary Table S1). In addition, BMI was not observed to interact with the arm, sex, race, and cigarette smoking status (*P* > 0.05).

### Age-specific BMI, average BMI, weight change, and CRC risk

The age-specific BMI analyses suggested that being overweight and obese at various stages of adulthood was more strongly associated with CRC risk, and the lowest HRs were obtained for the baseline measurement, which could be due to an unfavourable metabolic profile (mean age at baseline = 64.3).^11^ Specifically, compared with subjects who were never overweight/obese, subjects who first exceeded the threshold of 25 kg m^−2^ at age 20 had a higher CRC risk (HR = 1.28, 95% CI = 1.11–1.48) (Supplementary Table S2). When modeled continuously, subjects whose average BMI across the three time periods were classified as overweight or obese had an increased CRC risk (HR = 1.22, 1.18, and 1.21 per 5 kg m^−2^ increase) compared with normal-BMI subjects (Supplementary Table S3). A body weight gain of ≥15 kg between 20 and 50 years of age (HR = 1.34, 95% CI = 1.18–1.52) and baseline (HR = 1.24, 95% CI = 1.08–1.43) conferred a significant increase in CRC risk. Adjusting for the initial weight did not significantly affect the results (Supplementary Table S4).

### BMI trajectories and CRC risk

**Fig. 1:**
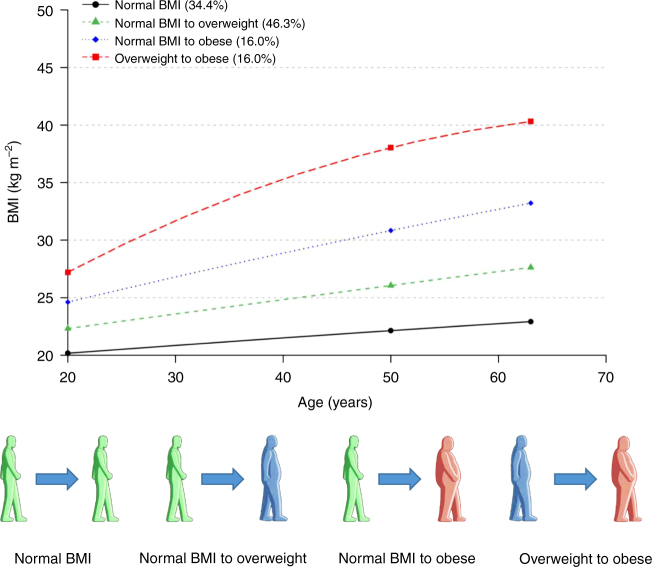
Latent class growth model of BMI trajectories in the PLCO study. Each trajectory was calculated at any of the three analysed age points (ages of 20 years, 50 years, and baseline). BMI body mass index

Age-specific BMI trajectories are illustrated in Fig. 1. Compared with subjects that had a normal BMI throughout their adult lifespan, the subjects who progressed from a normal BMI at the onset of adulthood to an obese BMI at baseline had an increased CRC risk (HR = 1.18, 95% CI = 1.03–1.35) (Supplementary Table S5). CRC risk showed a borderline increase in subjects who were overweight (HR = 1.27, 95% CI = 0.99–1.64) at the onset of adulthood and became obese at baseline. This result could be due to the limited sample size in the overweight and obese groups because the *P*_trend_ (*P*_trend_ = 0.005) is statistically significant.

Furthermore, stratified analyses by demographic characteristics (randomisation arm, sex, family history of CRC, and cigarette smoking status) showed little connection to BMI and CRC risk, and a subanalysis by CRC characteristics (location, stage, and grade) did not reveal significant associations between BMI and CRC risk (Supplementary Tables S6-S11).

## Discussion

The results obtained for age-specific BMI, average BMI, body weight change, and BMI trajectory indicate a significant positive relationship between increased BMI and CRC risk if the subjects became overweight/obese at the onset of adulthood. Prior studies performed a dose–response analysis at a single time point in adulthood to demonstrate that an elevated BMI increases CRC risk.^12^ However, the weight of a subject might change over his/her adult life, and weight as a dynamic variable might play a critical role in the onset and progression of CRC.^13^ Therefore, our study expanded on prior studies assessing the association of BMI metrics across the adult lifespan with CRC risk.

Based on the abovementioned study, a BMI trajectory analysis was performed in this study, and the findings revealed that subjects with a normal or overweight BMI at the onset of adulthood that became obese by baseline have a higher CRC risk, suggesting that being overweight before adulthood could have biological effects on growth or be caused by an increased duration of excess weight.^14^

To our knowledge, a cohort study reduces the probability of recall bias, and this study might be suitable for examining the association between different BMI metrics over a subject’s lifespan and CRC risk.^1,7,10^ These findings may help young adults reduce CRC risk by focusing on body weight loss. We relied on self-reported body weight and height, which potentially introduced misclassifications in our assessment of BMI and might have limited the ability to investigate the association in this study.^15^ Another limitation is that we lack data for other measures of excess body fat, which might provide greater insight into the connection between excess body fat and CRC risk. Additionally, underlying diseases or lifestyle characteristics may cause weight loss, which affects the association between BMI trajectory and CRC risk.

In summary, this study suggests that becoming overweight/obese in early adulthood might affect the incidence of CRC and that body weight gain over the adult lifespan is associated with a slightly elevated CRC risk. These findings offer a significant strategy to aid the primary prevention of CRC in subjects with a high BMI starting at early adulthood.

## Electronic supplementary material

Supplementary materials
