# Supplementary material for: Body mass index (BMI) trajectories and risk of colorectal cancer in the PLCO cohort
Source: Br J Cancer. 2018 Jun 6;119(1):130–2. doi: 10.1038/s41416-018-0121-y (PMC6035226; doi:10.1038/s41416-018-0121-y)
Supplement: Supplementary file 1 — Supplementary materials [file 41416_2018_121_MOESM1_ESM.doc]

**Materials**

Details of this study’s design, ascertainment of CRC, assessment of BMI and statistical analysis are presented below.

**Study population**

The PLCO cohort study randomly recruited 154,897 individuals aged 49-78 years from 10 USA research centers from 1993 to 2001. The PLCO cohort study has been described previously [^1-4^](#_ENREF_1). In brief, individuals were randomly assigned to the intervention group or the control group via flexible sigmoidoscopy screening at baseline and again during follow-up. Furthermore, participants with suspected abnormal findings (sigmoidoscopically visualized masses or polypoid lesions) were followed up with an endoscopic and histopathologic evaluation by their health-care providers. All the individuals provided written informed consent. Based on the original selection criteria for the PLCO study, we excluded 15,668 individuals in the present study for the following reasons: personal history of cancer prior to trial entry (n = 11,803) or missing (n = 3,490) or BMI values (< 15 or > 50 kg m^-2^, n = 375) at any of the three analyzed age points. Exclusion of these individuals resulted in the inclusion of 139,229 subjects, which included 70,541 subjects in the intervention arm and 68,688 in the control arm.

**Ascertainment of colorectal cancer**

In total, 2,031 CRC diagnoses (1,196 men and 835 women) were confirmed during a median follow-up time of 11.9 years. According to the International Classification of Diseases for Oncology Second Edition (ICD-O-2), the CRC characteristics were described by the anatomical position (left-sided CRC and right-sided CRC)[^5-7^](#_ENREF_5), pathologic stage (early stage and advanced stage) and grade (well differentiated, moderately differentiated and poorly differentiated).

**Assessment of BMI**

The baseline risk factor questionnaires included two questions pertaining to BMI data: (1) How tall are you? (a height less than 48 inches was out of range; for female participants, a height greater than 78 inches was out of range; and for male participants, a height greater than 84 inches was out of range) and (2) What is or was your weight at the three analyzed age points? (ages of 20 years, 50 years and baseline; for all participants, a weight less than 60 pounds was out of range). BMI metrics were ascertained at the three analyzed age points, namely, at the onset of adulthood (age 20 years), at mid-adulthood (age 50 years), and at the age at baseline (trial entry). We also divided the BMI metrics across three time periods into three categories: 20 to 50 years of age, 20 years of age to baseline and 50 years of age to baseline. Furthermore, we assessed the age of each individual at which his/her BMI first exceeded 25 kg m^-2^, because when the BMI > 25 kg m^-2^ means overweight or obese [^8^](#_ENREF_8).

**Statistical analysis**

CRC was ascertained through completion of baseline questionnaires until CRC diagnosis, death, or loss to follow-up (whichever came first). We used continuous variables to conduct tests of linear trend. The proportional hazards assumption was not violated as determined by visualization of Schoenfeld residuals plots (*P* ≥ 0.05) (**Supplementary Figure S1**) . Candidate confounders included arm, sex, race, family history of CRC, education, marital status, cigarette smoking status, aspirin and NSAID use, diabetes, physical activity, intakes of alcohol, fruit/vegetables and meat , and all of the variables were collected at baseline. We adjusted for variables that, when added to adjusted model, changed the matched HR by ≥10% [^12^](#_ENREF_12); arm (intervention and control), sex (male and female), family history of CRC (yes or no), cigarette smoking status (yes or no) and race (white, black, Hispanic or Asian) met the criterion and were defined as established variables, and study center was considered a potential confounder. A multivariable statistical analysis was conducted with adjustment for established and potential confounding factors. Furthermore, we additionally adjusted for height (cm) in models of weight change. Additionally, the normal BMI was included in the reference category in all the models. We used a likelihood ratio test to examine the interactions of BMI with the randomization arm, sex, race and cigarette smoking status and thus to determine the feasibility of the correlations between BMI and CRC risk.

The latent class growth model (LCGM) was fitted using linear and quadratic polynomials with three to five trajectory categories (individuals per trajectory ≥1%). Moreover, we selected the model with the highest number of fitting categories using the Bayesian Information Criterion (BIC) method and the average posterior probability (AvePP) of each trajectory [^13^](#_ENREF_13).

In the sensitivity analyses, we examined whether weight gain changed the CRC risk beyond that obtained with the models adjusted for the initial weight at the investigated age points during adulthood [^14^](#_ENREF_14).

**References**

1. Prorok PC, Andriole GL, Bresalier RS, Buys SS, Chia D, Crawford ED, et al. Design of the Prostate, Lung, Colorectal and Ovarian (PLCO) Cancer Screening Trial. Control Clin Trials. 2000;21(6 Suppl):273s-309s. Epub 2001/02/24.

2. Gohagan JK, Prorok PC, Greenwald P, Kramer BS. The PLCO Cancer Screening Trial: Background, Goals, Organization, Operations, Results. Rev Recent Clin Trials. 2015;10(3):173-80. Epub 2015/08/05.

3. Kitahara CM, Berndt SI, de Gonzalez AB, Coleman HG, Schoen RE, Hayes RB, et al. Prospective investigation of body mass index, colorectal adenoma, and colorectal cancer in the prostate, lung, colorectal, and ovarian cancer screening trial. Journal of clinical oncology : official journal of the American Society of Clinical Oncology. 2013;31(19):2450-9. Epub 2013/05/30.

4. Dominianni C, Huang WY, Berndt S, Hayes RB, Ahn J. Prospective study of the relationship between coffee and tea with colorectal cancer risk: the PLCO Cancer Screening Trial. British journal of cancer. 2013;109(5):1352-9. Epub 2013/08/03.

5. Lee YM, Law WL, Chu KW, Poon RT. Emergency surgery for obstructing colorectal cancers: a comparison between right-sided and left-sided lesions. J Am Coll Surg. 2001;192(6):719-25.

6. Meza R, Jeon J, Renehan AG, Luebeck EG. Colorectal cancer incidence trends in the United States and United kingdom: evidence of right- to left-sided biological gradients with implications for screening. Cancer research. 2010;70(13):5419-29.

7. Loree JM, Pereira AAL, Lam M, Willauer AN, Raghav K, Dasari A, et al. Classifying Colorectal Cancer by Tumor Location Rather than Sidedness Highlights a Continuum in Mutation Profiles and Consensus Molecular Subtypes. Clin Cancer Res. 2017;27:1078-0432.

8. Adams KF, Leitzmann MF, Ballard-Barbash R, Albanes D, Harris TB, Hollenbeck A, et al. Body mass and weight change in adults in relation to mortality risk. American journal of epidemiology. 2014;179(2):135-44.

9. Schoenfeld D. Partial residuals for the proportional hazards regression model. Biometrika. 1982;69(1):239-41.

10. Petrick JL, Kelly SP, Liao LM, Freedman ND, Graubard BI, Cook MB. Body weight trajectories and risk of oesophageal and gastric cardia adenocarcinomas: a pooled analysis of NIH-AARP and PLCO Studies. British journal of cancer. 2017;116(7):951-9. Epub 2017/02/15.

11. Kelly SP, Graubard BI, Andreotti G, Younes N, Cleary SD, Cook MB. Prediagnostic Body Mass Index Trajectories in Relation to Prostate Cancer Incidence and Mortality in the PLCO Cancer Screening Trial. Journal of the National Cancer Institute. 2017;109(3):1-9. Epub 2017/04/05.

12. Kernan WN, Viscoli CM, Brass LM, Broderick JP, Brott T, Feldmann E, et al. Phenylpropanolamine and the risk of hemorrhagic stroke. N Engl J Med. 2000;343(25):1826-32.

13. Andruff H, Carraro N, Thompson A, Gaudreau P. Latent Class Growth Modelling: A Tutorial. Tutorials in Quantitative Methods for Psychology. 2009;5(1).

14. Tu YK, Gilthorpe MS. Revisiting the relation between change and initial value: a review and evaluation. Stat Med. 2007;26(2):443-57.


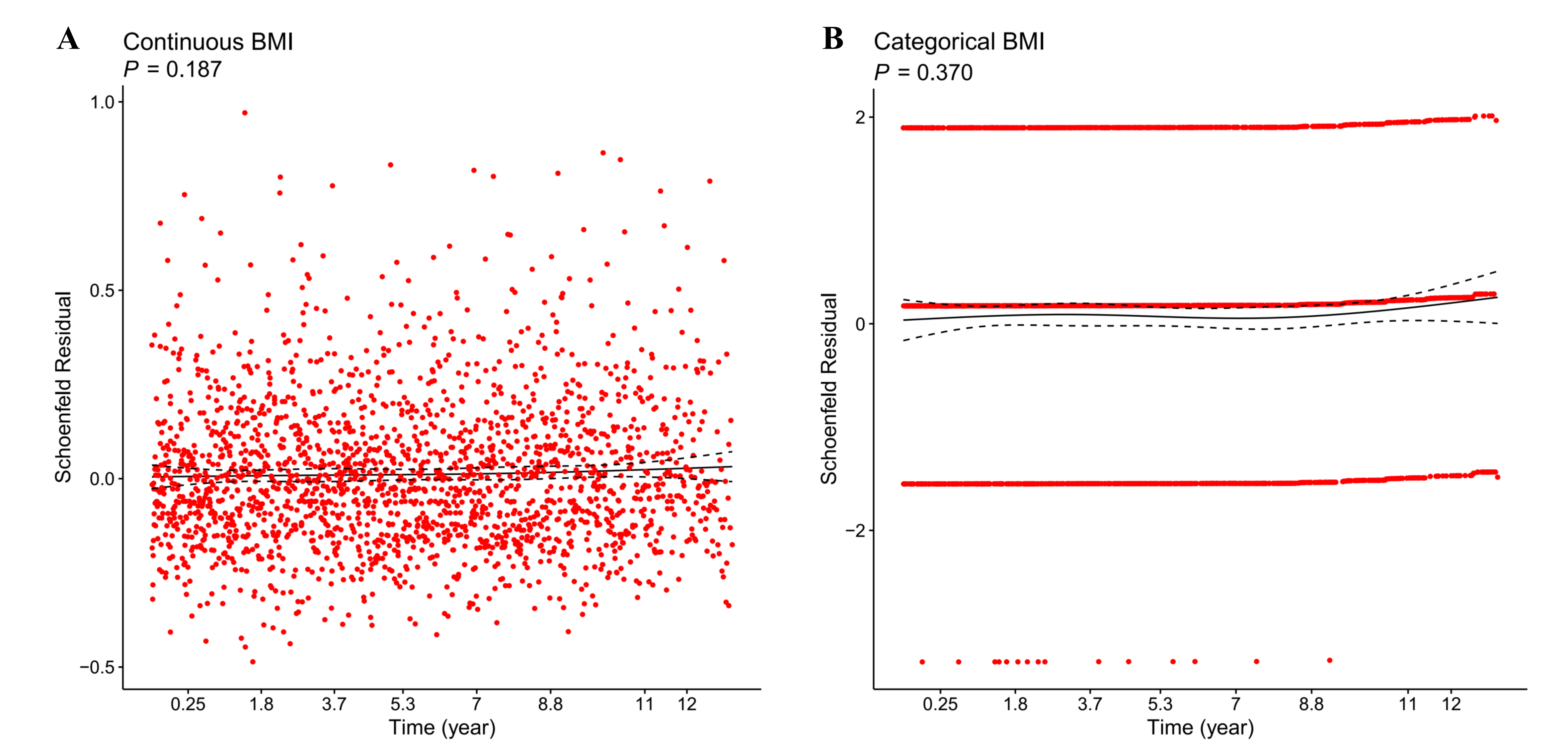


Figure S1. Schoenfeld residual for continuous and categorical BMI values versus survival time

Table S1. Characteristics of the subjects in the PLCO study

| Variables | Total (N = 139,229) | Cases (N = 2,031) |
| --- | --- | --- |
| Arm, No (%) |  |  |
| Intervention | 70,541 (50.67) | 901 (44.36) |
| Control | 68,688 (49.33) | 1,130 (55.64) |
| Age at trial entry |  |  |
| Mean±SD | 62.6±5.3 | 64.3±5.3 |
| Age at diagnosis/exit trial |  |  |
| Mean±SD | 73.5±5.9 | 69.8±6.4 |
| Sex, No (%) |  |  |
| Male | 70,344 (50.52) | 1,196 (58.89) |
| Female | 68,885 (49.48) | 835 (41.11) |
| Race, No (%) |  |  |
| White | 123,337 (88.59) | 1,793 (88.28) |
| Black | 6,963 (5.00) | 121 (5.96) |
| Hispanic | 2,576 (1.85) | 31 (1.53) |
| Asian | 5,181 (3.72) | 75 (3.69) |
| Other | 1,172 (0.84) | 11 (0.54) |
| Family history of CRC |  |  |
| Yes | 13,901 (10.33) | 256 (13.25) |
| No | 120,621 (89.67) | 1,676 (86.75) |
| Missing | 4,707 | 99 |
| Height (cm) |  |  |
| Mean±SD | 170.5±9.9 | 172.0±9.9 |
| BMI at age 20y, mean±SD, kg m^-2^ | 22.1±3.0 | 22.4±3.2 |
| BMI at age 50y, mean±SD, kg m^-2^ | 25.9±4.2 | 26.2±4.2 |
| BMI at baseline, mean±SD, kg m^-2^ | 27.3±4.7 | 27.5±4.6 |
| Cigarette smoking status |  |  |
| Yes | 74,969 (53.85) | 1,197 (58.94) |
| No | 64,244 (46.15) | 834 (41.06) |
| Missing | 16 | 0 |
| Location |  |  |
| Left-sided CRC |  | 890 (50.42) |
| Right-sided CRC |  | 875 (49.58) |
| Missing |  | 266 |
| CRC stage |  |  |
| Early stage |  | 1191 (58.93) |
| Advanced stage |  | 830 (41.07) |
| Missing |  | 10 |
| Grade |  |  |
| Well |  | 207 (11.02) |
| Moderate |  | 1,286 (68.48) |
| Poor |  | 385 (20.50) |
| Missing |  | 153 |

Abbreviations: SD, standard deviation.

Left-side CRC: rectum, sigmoid colon, descending colon and splenic flexure of colon.

Right-side CRC: transverse colon, ascending colon and cecum.

Early stage: stage I/II.
Advanced stage: stage III/IV

Table S2. Association between age-specific BMI and CRC incidence in the PLCO study

| Age-specific BMI | Cases (N = 2,031) | HR (95% CI^a^) |
| --- | --- | --- |
| BMI, age 20 |  |  |
| < 18.5 | 154 | 1.08 (0.91-1.27) |
| ≥ 18.5-25 | 1,505 | Reference |
| ≥ 25-30 | 329 | 1.26 (1.11-1.43) |
| ≥ 30 | 43 | 1.43 (1.04-1.95) |
| *P*trend |  | 0.003 |
| Continuous, per 5 kg m^-2^ |  | 1.14 (1.05-1.24) |
| BMI, age 50 |  |  |
| < 18.5 | 16 | 1.27 (0.76-2.12) |
| ≥ 18.5-25 | 833 | Reference |
| ≥ 25-30 | 860 | 1.22 (1.10-1.35) |
| ≥ 30 | 322 | 1.63 (1.43-1.87) |
| *P*trend |  | < 0.001 |
| Continuous, per 5 kg m^-2^ |  | 1.25 (1.18-1.34) |
| BMI at baseline |  |  |
| < 18.5 | 15 | 1.27 (0.74-2.21) |
| ≥ 18.5-25 | 596 | Reference |
| ≥ 25-30 | 913 | 1.12 (1.00-1.24) |
| ≥ 30 | 507 | 1.17 (1.03-1.32) |
| *P*trend |  | 0.019 |
| Continuous, per 5 kg m^-2^ |  | 1.07 (1.01-1.14) |
| Time when BMI first exceeded  25 kg m^-2^ |  |  |
| Never | 501 | Reference |
| Age 20 y | 372 | 1.28 (1.11-1.48) |
| Age 50 y | 833 | 1.17 (1.04-1.32) |
| Baseline age | 325 | 1.20 (1.04-1.38) |

Abbreviations: HR, hazard ratio; CI, confidence intervals.
^a^ Adjusted for randomization arm (intervention or control), sex (male or female), study center, race (white, black, Hispanic or Asian), family history of CRC (yes or no) and cigarette smoking status (yes or no).

Table S3. Association between average BMI and CRC incidence in the PLCO study

| Average BMI | Cases (N = 2,031) | HR (95% CI^a^) |
| --- | --- | --- |
| Age 20 and 50 |  |  |
| < 18.5 | 22 | 1.02 (0.66-1.58) |
| ≥ 18.5-25 | 1,301 | Reference |
| ≥ 25-30 | 598 | 1.22 (1.10-1.35) |
| ≥ 30 | 110 | 1.53 (1.25-1.87) |
| *P*trend |  | < 0.001 |
| Continuous, per 5 kg m^-2^ |  | 1.22 (1.13-1.31) |
| Age 20 and baseline |  |  |
| < 18.5 | 13 | 0.88 (0.48-1.59) |
| ≥ 18.5-25 | 1,132 | Reference |
| ≥ 25-30 | 734 | 1.15 (1.05-1.27) |
| ≥ 30 | 152 | 1.45 (1.22-1.72) |
| *P*trend |  | < 0.001 |
| Continuous, per 5 kg m^-2^ |  | 1.18 (1.10-1.27) |
| Age 50 and baseline |  |  |
| < 18.5 | 12 | 1.31 (0.70-2.46) |
| ≥ 18.5-25 | 685 | Reference |
| ≥ 25-30 | 944 | 1.20 (1.08-1.33) |
| ≥ 30 | 390 | 1.50 (1.31-1.70) |
| *P*trend |  | < 0.001 |
| Continuous, per 5 kg m^-2^ |  | 1.21 (1.14-1.29) |

Abbreviations: HR, hazard ratio; CI, confidence intervals.
^a^ Adjusted for randomization arm (intervention or control), sex (male or female), study center, race (white, black, Hispanic or Asian), family history of CRC (yes or no) and cigarette smoking status (yes or no).

Table S4. Association of body weight change with CRC incidence in the PLCO study

| Feature | Cases (N = 2,031) | HR (95% CI) |
| --- | --- | --- |
| Weight change, kg |  |  |
| Age 20-50^a^ |  |  |
| ≤ -2 | 110 | 1.07 (0.87-1.34) |
| > -2 to < 5 | 456 | Reference |
| ≥ 5 to < 15 | 876 | 1.08 (0.97-1.22) |
| ≥ 15 | 589 | 1.34 (1.18-1.52) |
| *P*trend |  | < 0.001 |
| Continuous, per 5 kg |  | 1.15 (1.10-1.22) |
| Age 20 to baseline^a^ |  |  |
| ≤ -2 | 128 | 1.22 (0.98-1.52) |
| > -2 to < 5 | 268 | Reference |
| ≥ 5 to < 15 | 689 | 1.08 (0.94-1.26) |
| ≥ 15 | 946 | 1.24 (1.08-1.43) |
| *P*trend |  | 0.019 |
| Continuous, per 5 kg |  | 1.06 (1.01-1.12) |
| Age 50 to baseline^a^ |  |  |
| ≤ -2 | 392 | 1.16 (0.94-1.31) |
| > -2 to < 5 | 883 | Reference |
| ≥ 5 to < 15 | 590 | 1.05 (0.94-1.16) |
| ≥ 15 | 166 | 1.01 (0.85-1.20) |
| *P*trend |  | 0.242 |
| Continuous, per 5 kg |  | 0.97 (0.92-1.02) |
| Adjusted for initial weight (kg)^b^ | | |
| Age 20-50 |  |  |
| ≤ -2 | 110 | 0.96（0.77-1.21） |
| > -2 to < 5 | 456 | Reference |
| ≥ 5 to < 15 | 876 | 1.11（0.99-1.25） |
| ≥ 15 | 589 | 1.39 (1.22-1.58) |
| Continuous, per 5 kg |  | 1.15 (1.09-1.22) |
| Age 20 to baseline |  |  |
| ≤ -2 | 128 | 1.12 (0.90-1.40) |
| > -2 to < 5 | 268 | Reference |
| ≥ 5 to < 15 | 689 | 1.10 (0.96-1.28) |
| ≥ 15 | 946 | 1.29 (1.12-1.48) |
| Continuous, per 5 kg |  | 1.09 (1.04-1.15) |
| Age 50 to baseline |  |  |
| ≤ -2 | 392 | 1.04 (0.91-1.18) |
| > -2 to < 5 | 883 | Reference |
| ≥ 5 to < 15 | 590 | 1.02 (0.92-1.13) |
| ≥ 15 | 166 | 0.95 (0.80-1.13) |
| Continuous, per 5 kg |  | 0.98 (0.94-1.04) |

Abbreviations: HR, hazard ratio; CI, confidence intervals.
^a^ Adjusted for randomization arm (intervention or control), sex (male or female), study center, race (white, black, Hispanic or Asian), family history of CRC (yes or no), cigarette smoking status (yes or no) and height (cm).

^b^ Adjusted for randomization arm (intervention or control), sex (male or female), study center, race (white, black, Hispanic or Asian), family history of CRC (yes or no), cigarette smoking status (yes or no), weight (kg) at the beginning of the time period and height (cm).

Table S5. Association between BMI trajectories and CRC incidence in the PLCO study

| BMI trajectory | Total (N = 139,229) | |
| --- | --- | --- |
|  | Cases (N = 2,031) | HR (95% CI^a^) |
| Normal BMI | 608 | Reference |
| Normal BMI to overweight | 1,008 | 1.11 (0.99-1.23) |
| Normal BMI to obese | 345 | 1.18 (1.03-1.35) |
| overweight BMI to obese | 70 | 1.27 (0.99-1.64) |
| *P*_trend_ |  | 0.005 |

Abbreviations: HR, hazard ratio; CI, confidence intervals.
^a^ Adjusted for randomization arm (intervention or control), sex (male or female), study center, race (white, black, Hispanic or Asian), family history of CRC (yes or no) and cigarette smoking status (yes or no).

Table S6. Association of demographic characteristics and age-specific BMI in relation to CRC incidence in the PLCO study

| Age-specific BMI | Randomization arm | | | |  | Sex | | | |  | Family history of CRC | | | |  | Cigarette smoking status | | | |
| --- | --- | --- | --- | --- | --- | --- | --- | --- | --- | --- | --- | --- | --- | --- | --- | --- | --- | --- | --- |
|  | Intervention (N = 901) | | Control (N = 1,130) | |  | Male (N = 1,196) | | Female (N = 835) | |  | No (N = 1,676) | | Yes (N = 256) | |  | No (N = 834) | | Yes (N = 1,197) | |
|  | Case | HR (95% CI^a^) | Case | HR (95% CI^a^) |  | Case | HR (95% CI^a^) | Case | HR (95% CI^a^) |  | Case | HR (95% CI^a^) | Case | HR (95% CI^a^) |  | Case | HR (95% CI^a^) | Case | HR (95% CI^a^) |
| BMI, age 20 |  |  |  |  |  |  |  |  |  |  |  |  |  |  |  |  |  |  |  |
| < 18.5 | 65 | 0.99 (0.76-1.29) | 89 | 1.14 (0.92-1.43) |  | 59 | 1.10 (0.84-1.44) | 95 | 1.06 (0.86-1.32) |  | 130 | 1.06 (0.88-1.27) | 22 | 1.20 (0.77-1.87) |  | 56 | 0.90 (0.68-1.19) | 98 | 1.22 (0.98-1.50) |
| ≥ 18.5-25 | 673 | Reference | 832 | Reference |  | 835 | Reference | 670 | Reference |  | 1,242 | Reference | 186 | Reference |  | 632 | Reference | 873 | Reference |
| ≥ 25-30 | 144 | 1.29 (1.07-1.56) | 185 | 1.24 (1.05-1.46) |  | 275 | 1.31 (1.14-1.51) | 54 | 1.08 (0.81-1.45) |  | 269 | 1.23 (1.08-1.41) | 43 | 1.49 (1.06-2.09) |  | 134 | 1.32 (1.08-1.61) | 195 | 1.22 (1.04-1.44) |
| ≥ 30 | 19 | 1.38 (0.86-2.20) | 24 | 1.48 (0.97-2.26) |  | 27 | 1.41 (0.95-2.10) | 16 | 1.47 (0.88-2.46) |  | 35 | 1.43 (1.02-2.01) | 5 | － |  | 12 | 1.03 (0.57-1.87) | 31 | 1.67 (1.15-2.42) |
| *P*_trend_ |  | 0.013 |  | 0.070 |  |  | 0.001 |  | 0.527 |  |  | < 0.001 |  | 0.159 |  |  | 0.017 |  | 0.041 |
| Continuous, per 5 kg/m^2^ |  | 1.18 (1.04-1.34) |  | 1.11 (0.99-1.25) |  |  | 1.19 (1.08-1.33) |  | 1.05 (0.91-1.21) |  |  | 1.14 (1.04-1.25) |  | 1.19 (0.94-1.51) |  |  | 1.18 (1.03-1.35) |  | 1.11 (1.00-1.24) |
| BMI, age 50 |  |  |  |  |  |  |  |  |  |  |  |  |  |  |  |  |  |  |  |
| < 18.5 | 5 | － | 11 | 1.69 (0.93-3.08) |  | 6 | 2.01 (0.83-4.86) | 10 | 1.09 (0.58-2.03) |  | 14 | 1.36 (0.80-2.31) | 1 | － |  | 9 | 1.62 (0.80-2.37) | 7 | 1.03 (0.49-2.18) |
| ≥ 18.5-25 | 381 | Reference | 452 | Reference |  | 381 | Reference | 452 | Reference |  | 701 | Reference | 93 | Reference |  | 340 | Reference | 493 | Reference |
| ≥ 25-30 | 360 | 1.13 (0.97-1.31) | 500 | 1.30 (1.13-1.48) |  | 598 | 1.17 (1.03-1.34) | 262 | 1.32 (1.13-1.54) |  | 697 | 1.16 (1.04-1.29) | 121 | 1.67 (1.26-2.21) |  | 360 | 1.34 (1.15-1.57) | 500 | 1.34 (1.00-1.30) |
| ≥ 30 | 155 | 1.67 (1.38-2.03) | 167 | 1.60 (1.33-1.92) |  | 211 | 1.67 (1.41-1.99) | 111 | 1.56 (1.26-1.93) |  | 264 | 1.57 (1.36-1.82) | 41 | 2.09 (1.44-3.03) |  | 125 | 1.55 (1.25-1.92) | 197 | 1.69 (1.42-2.00) |
| *P*_trend_ |  | < 0.001 |  | < 0.001 |  |  | < 0.001 |  | < 0.001 |  |  | < 0.001 |  | < 0.001 |  |  | < 0.001 |  | < 0.001 |
| Continuous, per 5 kg/m^2^ |  | 1.21 (1.15-1.39) |  | 1.25 ( 1.14-1.36) |  |  | 1.26 (1.16-1.38) |  | 1.25 (1.14-1.38) |  |  | 1.22 (1.14-1.31) |  | 1.48 (1.24-1.76) |  |  | 1.25 (1.13-1.37) |  | 1.26 (1.16-1.37) |
| BMI at baseline |  |  |  |  |  |  |  |  |  |  |  |  |  |  |  |  |  |  |  |
| < 18.5 | 3 | － | 12 | 2.09 (0.94-3.62) |  | 4 | － | 11 | 1.34 (0.71-2.51) |  | 11 | 1.27 (0.70-2.32) | 2 | － |  | 9 | 1.88 (0.93-3.80) | 6 | 0.86 (0.35-2.07) |
| ≥ 18.5-25 | 267 | Reference | 329 | Reference |  | 291 | Reference | 305 | Reference |  | 497 | Reference | 66 | Reference |  | 252 | Reference | 344 | Reference |
| ≥ 25-30 | 393 | 1.05 (0.89-1.23) | 520 | 1.17 (1.01-1.35) |  | 607 | 1.10 (0.95-1.27) | 306 | 1.14 (0.97-1.34) |  | 753 | 1.09 (0.97-1.22) | 119 | 1.33 (0.98-1.80) |  | 372 | 1.18 (0.99-1.40) | 541 | 1.07 (0.93-1.23) |
| ≥ 30 | 238 | 1.18 (0.99-1.42) | 269 | 1.15 (0.97-1.36) |  | 294 | 1.16 (0.98-1.38) | 213 | 1.18 (0.98-1.41) |  | 415 | 1.13 (0.99-1.29) | 69 | 1.42 (1.01-2.00) |  | 201 | 1.20 (0.99-1.45) | 306 | 1.14 (0.97-1.34) |
| *P*_trend_ |  | 0.188 |  | 0.621 |  |  | 0.081 |  | 0.094 |  |  | 0.085 |  | 0.046 |  |  | 0.103 |  | 0.09 |
| Continuous, per 5 kg/m^2^ |  | 1.10 (1.01-1.20) |  | 1.05 (0.97-1.14) |  |  | 1.08 (0.99-1.17) |  | 1.08 (0.99-1.17) |  |  | 1.06 (0.99-1.13) |  | 1.18 (1.00-1.39) |  |  | 1.08 (0.99-1.18) |  | 1.07 (0.99-1.16) |
| Time when BMI first exceeded 25 kg/m^2^ |  |  |  |  |  |  |  |  |  |  |  |  |  |  |  |  |  |  |  |
| Never | 223 | Reference | 278 | Reference |  | 224 | Reference | 277 | Reference |  | 420 | Reference | 57 | Reference |  | 213 | Reference | 288 | Reference |
| Age 20y | 163 | 1.28 (1.04-1.59) | 209 | 1.27 (1.05-1.54) |  | 302 | 1.29 (1.08-1.55) | 70 | 1.21 (0.92-1.59) |  | 304 | 1.23 (1.06-1.44) | 48 | 1.64 (1.10-2.45) |  | 146 | 1.39 (1.11-1.74) | 226 | 1.20 (1.00-1.44) |
| Age 50y | 362 | 1.13 (0.95-1.35) | 471 | 1.20 (1.03-1.41) |  | 519 | 1.13 (0.96-1.33) | 314 | 1.25 (1.06-1.47) |  | 675 | 1.13 (1.00-1.28) | 115 | 1.45 (1.08-2.06) |  | 349 | 1.29 (1.08-1.53) | 484 | 1.09 (0.94-1.27) |
| Baseline age | 153 | 1.22 (0.99-1.51) | 172 | 1.17 (0.97-1.43) |  | 151 | 1.26 (0.92-1.56) | 174 | 1.15 (0.95-1.40) |  | 277 | 1.21 (1.04-1.41) | 36 | 1.03 (0.72-1.65) |  | 126 | 1.21 (0.97-1.52) | 199 | 1.18 (0.98-1.42) |

Abbreviations: HR, hazard ratio; CI, confidence intervals.

Left-side CRC: rectum, sigmoid colon, descending colon and splenic flexure of colon.

Right-side CRC: transverse colon, ascending colon and cecum.

Early stage: stage I/II.
Advanced stage: stage III/IV.
^a^ Adjusted for randomization arm (intervention or control), sex (male or female), study center, race (white, black, Hispanic or Asian), family history of CRC (yes or no) and cigarette smoking status (yes or no).

Table S7. Association of CRC characteristics and age-specific BMI in relation to CRC incidence in the PLCO study

| Age-specific BMI | Location | | | |  | Stage | | | |  | Grade | | | | | |
| --- | --- | --- | --- | --- | --- | --- | --- | --- | --- | --- | --- | --- | --- | --- | --- | --- |
|  | Left-sided (N = 890) | | Right-sided (N = 875) | |  | Early stage (N = 1,191) | | Advanced stage (N = 830) | |  | Well (N = 207) | | Moderate (N = 1,286) | | Poor (N = 385) | |
|  | Case | HR (95% CI^a^) | Case | HR (95% CI^a^) |  | Case | HR (95% CI^a^) | Case | HR (95% CI^a^) |  | Case | HR (95% CI^a^) | Case | HR (95% CI^a^) | Case | HR (95% CI^a^) |
| BMI, age 20 |  |  |  |  |  |  |  |  |  |  |  |  |  |  |  |  |
| < 18.5 | 65 | 1.21 (0.93-1.57) | 76 | 0.94 (0.74-1.20) |  | 96 | 1.11 (0.89-1.38) | 57 | 0.96 (0.73-1.27) |  | 15 | 0.82 (0.47-1.40) | 85 | 1.11 (0.89-1.39) | 39 | 1.06 (0.75-1.49) |
| ≥ 18.5-25 | 667 | Reference | 625 | Reference |  | 871 | Reference | 628 | Reference |  | 154 | Reference | 960 | Reference | 283 | Reference |
| ≥ 25-30 | 142 | 1.06 (0.88-1.29) | 154 | 1.20 (1.00-1.44) |  | 201 | 1.15 (0.98-1.35) | 125 | 1.16 (0.94-1.43) |  | 35 | 0.81 (0.53-1.20) | 213 | 1.39 (1.19-1.63) | 56 | 0.85 (0.62-1.17) |
| ≥ 30 | 16 | 1.30 (0.77-2.18) | 20 | 1.22 (0.77-1.94) |  | 23 | 1.23 (0.79-1.90) | 20 | 1.39 (0.88-2.21) |  | 3 | － | 28 | 1.43 (0.96-2.14) | 7 | 0.83 (0.39-1.78) |
| *P*_trend_ |  | 0.939 |  | 0.049 |  |  | 0.374 |  | 0.069 |  |  | 0.908 |  | 0.001 |  | 0.280 |
| Continuous, per 5 kg m^-2^ |  | 1.01 (0.88-1.15) |  | 1.13 (1.00-1.28) |  |  | 1.06 (0.95-1.19) |  | 1.13 (0.99-1.30) |  |  | 1.02 (0.78-1.33) |  | 1.20 (1.07-1.34) |  | 0.90 (0.75-1.09) |
| BMI, age 50 |  |  |  |  |  |  |  |  |  |  |  |  |  |  |  |  |
| < 18.5 | 7 | 1.11 (0.52-1.36) | 7 | 0.82 (0.36-1.85) |  | 9 | 1.28 (0.63-2.59) | 7 | 1.05 (0.49-2.23) |  | 1 | － | 12 | 1.04 (0.57-1.90) | 1 | － |
| ≥ 18.5-25 | 361 | Reference | 369 | Reference |  | 492 | Reference | 338 | Reference |  | 71 | Reference | 542 | Reference | 161 | Reference |
| ≥ 25-30 | 379 | 1.14 (0.97-1.33) | 354 | 0.97 (0.83-1.14) |  | 504 | 1.08 (0.95-1.24) | 352 | 1.08 (0.92-1.26) |  | 95 | 1.09 (0.78-1.53) | 536 | 1.12 (0.99-1.27) | 168 | 1.10 (0.80-1.53) |
| ≥ 30 | 143 | 1.10 (0.89-1.35) | 145 | 1.42 (1.16-1.73) |  | 186 | 1.39 (1.16-1.66) | 133 | 1.37 (1.11-1.69) |  | 40 | 0.99 (0.66-1.48) | 196 | 1.52 (1.28-1.81) | 55 | 1.08 (0.86-1.34) |
| *P*_trend_ |  | 0.243 |  | 0.008 |  |  | < 0.001 |  | 0.009 |  |  | 0.923 |  | < 0.001 |  | 0.425 |
| Continuous, per 5 kg m^-2^ |  | 1.06 (0.96-1.17) |  | 1.14 (1.04-1.26) |  |  | 1.15 (1.05-1.25) |  | 1.14 (1.03-1.26) |  |  | 1.01 (0.83-1.23) |  | 1.20 (1.10-1.30) |  | 1.06 (0.91-1.24) |
| BMI at baseline |  |  |  |  |  |  |  |  |  |  |  |  |  |  |  |  |
| < 18.5 | 6 | 1.32 (0.54-3.21) | 7 | 2.88 (0.96-6.23) |  | 10 | 1.80 (0.96-3.40) | 5 | － |  | 1 | － | 11 | 1.68 (0.89-3.18) | 3 | － |
| ≥ 18.5-25 | 258 | Reference | 265 | Reference |  | 345 | Reference | 248 | Reference |  | 52 | Reference | 377 | Reference | 122 | Reference |
| ≥ 25-30 | 413 | 0.96 (0.82-1.13) | 389 | 1.10 (0.93-1.29) |  | 545 | 0.98 (0.85-1.13) | 365 | 1.16 (0.98-1.37) |  | 94 | 1.19 (0.83-1.72) | 585 | 1.04 (0.91-1.20) | 167 | 0.96 (0.74-1.23) |
| ≥ 30 | 213 | 0.93 (0.77-1.12) | 214 | 0.95 (0.78-1.14) |  | 291 | 0.95 (0.80-1.11) | 212 | 1.14 (0.94-1.38) |  | 60 | 0.80 (0.54-1.19) | 313 | 1.09 (0.93-1.27) | 93 | 0.95 (0.71-1.26) |
| *P*_trend_ |  | 0.362 |  | 0.403 |  |  | 0.319 |  | 0.237 |  |  | 0.173 |  | 0.444 |  | 0.527 |
| Continuous, per 5 kg m^-2^ |  | 0.96 (0.87-1.05) |  | 0.96 (0.88-1.05) |  |  | 0.96 (0.89-1.04) |  | 1.06 (0.96-1.17) |  |  | 0.88 (0.73-1.06) |  | 1.03 (0.95-1.11) |  | 0.96 (0.83-1.10) |
| Time when BMI first exceeded 25 kg m^-2^ |  |  |  |  |  |  |  |  |  |  |  |  |  |  |  |  |
| Never | 239 | Reference | 241 | Reference |  | 290 | Reference | 208 | Reference |  | 38 | Reference | 323 | Reference | 105 | Reference |
| Age 20y | 162 | 0.90 (0.73-1.11) | 178 | 1.03 (0.85-1.26) |  | 224 | 0.99 (0.82-1.19) | 145 | 1.26 (1.00-1.59) |  | 38 | 0.73 (0.46-1.17) | 241 | 1.13 (0.94-1.34) | 63 | 1.03 (0.73-1.45) |
| Age 50y | 377 | 0.96 (0.81-1.13) | 347 | 0.95 (0.81-1.13) |  | 480 | 0.97 (0.83-1.13) | 349 | 1.05 (0.88-1.26) |  | 99 | 0.98 (0.66-1.46) | 506 | 0.99 (0.85-1.14) | 164 | 1.06 (0.81-1.37) |
| Baseline age | 144 | 0.84 (0.68-1.04) | 140 | 1.11 (0.90-1.36) |  | 197 | 0.90 (0.75-1.10) | 128 | 1.17 (0.93-1.46) |  | 32 | 0.94 (0.57-1.55) | 216 | 1.02 (0.86-1.22) | 53 | 0.88 (0.62-1.25) |

Abbreviations: HR, hazard ratio; CI, confidence intervals.

Left-side CRC: rectum, sigmoid colon, descending colon and splenic flexure of colon.

Right-side CRC: transverse colon, ascending colon and cecum.

Early stage: stage I/II.
Advanced stage: stage III/IV.
^a^ Adjusted for randomization arm (intervention or control), sex (male or female), study center, race (white, black, Hispanic or Asian), family history of CRC (yes or no) and cigarette smoking status (yes or no).

Table S8. Association of demographic characteristics and average BMI in relation to CRC incidence in the PLCO study

| Average BMI | Randomization arm | | | |  | Sex | | | |  | Family history of CRC | | | |  | Cigarette smoking status | | | |
| --- | --- | --- | --- | --- | --- | --- | --- | --- | --- | --- | --- | --- | --- | --- | --- | --- | --- | --- | --- |
|  | Intervention (N = 901) | | Control (N = 1,130) | |  | Male (N = 1,196) | | Female (N = 835) | |  | No (N = 1,734) | | Yes (N = 264) | |  | No (N = 834) | | Yes (N = 1,179) | |
|  | Case | HR (95% CI) | Case | HR (95% CI) |  | Case | HR (95% CI) | Case | HR (95% CI) |  | Case | HR (95% CI) | Case | HR (95% CI) |  | Case | HR (95% CI) | Case | HR (95% CI) |
| Age 20 and 50 |  |  |  |  |  |  |  |  |  |  |  |  |  |  |  |  |  |  |  |
| < 18.5 | 8 | 0.78 (0.37-1.65) | 14 | 1.22 (0.72-2.07) |  | 7 | 1.41 (0.63-3.16) | 15 | 0.94 (0.56-1.57) |  | 17 | 0.94 (0.58-1.52) | 4 | － |  | 11 | 1.11 (0.59-2.08) | 11 | 0.98 (0.54-1.77) |
| ≥ 18.5-25 | 582 | Reference | 719 | Reference |  | 677 | Reference | 624 | Reference |  | 1,090 | Reference | 154 | Reference |  | 523 | Reference | 778 | Reference |
| ≥ 25-30 | 255 | 1.17 (1.00-1.37) | 343 | 1.26 (1.10-1.44) |  | 446 | 1.21 (1.07-1.37) | 152 | 1.23 (1.03-1.48) |  | 482 | 1.17 (1.05-1.31) | 82 | 1.57 (1.19-2.07) |  | 263 | 1.40 (1.20-1.64) | 335 | 1.10 (0.96-1.26) |
| ≥ 30 | 56 | 1.70 (1.28-2.26) | 54 | 1.39 (1.04-1.85) |  | 66 | 1.44 (1.11-1.87) | 44 | 1.71 (1.25-2.35) |  | 87 | 1.46 (1.17-1.82) | 16 | 2.09 (1.25-3.51) |  | 37 | 1.25 (0.88-1.77) | 73 | 1.71 (1.34-2.19) |
| *P*_trend_ |  | < 0.001 |  | < 0.001 |  |  | < 0.001 |  | < 0.001 |  |  | < 0.001 |  | < 0.001 |  |  | < 0.001 |  | < 0.001 |
| Continuous, per 5 kg m^-2^ |  | 1.24 (1.11-1.39) |  | 1.20 (1.08-1.33) |  |  | 1.20 (1.09-1.32) |  | 1.26 (1.11-1.42) |  |  | 1.18 (1.09-1.28) |  | 1.45 (1.19-1.78) |  |  | 1.24 (1.11-1.40) |  | 1.20 (1.08-1.32) |
| Age 20 and baseline |  |  |  |  |  |  |  |  |  |  |  |  |  |  |  |  |  |  |  |
| < 18.5 | 4 | － | 9 | 1.17 (0.58-2.36) |  | 6 | 1.74 (0.72-4.21) | 7 | 0.63 (0.28-1.41) |  | 10 | 0.92 (0.49-1.72) | 1 | － |  | 6 | 0.69 (0.26-1.85) | 7 | 1.07 (0.51-2.25) |
| ≥ 18.5-25 | 503 | Reference | 629 | Reference |  | 602 | Reference | 530 | Reference |  | 932 | Reference | 144 | Reference |  | 460 | Reference | 672 | Reference |
| ≥ 25-30 | 318 | 1.12 (0.97-1.30) | 416 | 1.17 (1.03-1.34) |  | 498 | 1.15 (1.01-1.30) | 236 | 1.17 (1.00-1.37) |  | 608 | 1.15 (1.04-1.28) | 90 | 1.16 (0.89-1.51) |  | 318 | 1.30 (1.12-1.51) | 416 | 1.05 (0.93-1.20) |
| ≥ 30 | 76 | 1.62 (1.27-2.07) | 76 | 1.31 (1.03-1.67) |  | 90 | 1.49 (1.19-1.37) | 62 | 1.40 (1.07-1.84) |  | 126 | 1.43 (1.19-1.72) | 21 | 1.58 (1.00-2.50) |  | 50 | 1.20 (0.89-1.62) | 102 | 1.60 (1.30-1.98) |
| *P*_trend_ |  | < 0.001 |  | 0.004 |  |  | < 0.001 |  | < 0.001 |  |  | < 0.001 |  | 0.044 |  |  | 0.002 |  | 0.001 |
| Continuous, per 5 kg m^-2^ |  | 1.22 (1.09-1.35) |  | 1.15 (1.05-1.27) |  |  | 1.18 (1.07-1.29) |  | 1.19 (1.07-1.33) |  |  | 1.17 (1.09-1.27) |  | 1.22 (1.01-1.48) |  |  | 1.20 (1.07-1.33) |  | 1.17 (1.06-1.28) |
| Age 50 and baseline |  |  |  |  |  |  |  |  |  |  |  |  |  |  |  |  |  |  |  |
| < 18.5 | 4 | － | 8 | 1.83 (0.86-3.87) |  | 5 | － | 7 | 1.01 (0.45-2.26) |  | 10 | 1.49 (0.80-2.79) | 0 | － |  | 6 | 1.52 (0.63-3.68) | 6 | 1.18 (0.49-2.86) |
| ≥ 18.5-25 | 308 | Reference | 377 | Reference |  | 330 | Reference | 355 | Reference |  | 579 | Reference | 73 | Reference |  | 285 | Reference | 400 | Reference |
| ≥ 25-30 | 407 | 1.16 (0.99-1.35) | 537 | 1.23 (1.07-1.41) |  | 619 | 1.15 (1.00-1.32) | 325 | 1.28 (1.10-1.49) |  | 767 | 1.14 (1.02-1.28) | 130 | 1.62 (1.21-2.17) |  | 394 | 1.31 (1.12-1.54) | 550 | 1.12 (0.98-1.29) |
| ≥ 30 | 182 | 1.53 (1.27-1.84) | 208 | 1.46 (1.23-1.75) |  | 242 | 1.54 (1.30-1.83) | 148 | 1.43 (1.17-1.74) |  | 320 | 1.43 (1.25-1.65) | 53 | 2.01 (1.38-2.82) |  | 149 | 1.41 (1.15-1.73) | 241 | 1.55 (1.31-1.83) |
| *P*_trend_ |  | < 0.001 |  | < 0.001 |  |  | < 0.001 |  | < 0.001 |  |  | < 0.001 |  | < 0.001 |  |  | < 0.001 |  | < 0.001 |
| Continuous, per 5 kg m^-2^ |  | 1.23 (1.12-1.35) |  | 1.19 (1.10-1.30) |  |  | 1.22 (1.12-1.33) |  | 1.21 (1.10-1.32) |  |  | 1.18 (1.10-1.26) |  | 1.43 (1.21-1.70) |  |  | 1.20 (1.09-1.32) |  | 1.23 (1.13-1.33) |

Abbreviations: HR, hazard ratio; CI, confidence intervals.
^a^ Adjusted for randomization arm (intervention or control), sex (male or female), study center, race (white, black, Hispanic or Asian), family history of CRC (yes or no) and cigarette smoking status (yes or no

Table S9. Association of CRC characteristics and average BMI in relation to CRC incidence in the PLCO study

| Average BMI | Location | | | |  | Stage | | | |  | Grade | | | | | |
| --- | --- | --- | --- | --- | --- | --- | --- | --- | --- | --- | --- | --- | --- | --- | --- | --- |
|  | Left-sided (N = 890) | | Right-sided (N = 875) | |  | Early stage (N = 1,191) | | Advanced stage (N = 830) | |  | Well (N = 207) | | Moderate (N = 1,286) | | Poor (N = 385) | |
|  | Case | HR (95% CI^a^) | Case | HR (95% CI^a^) |  | Case | HR (95% CI^a^) | Case | HR (95% CI^a^) |  | Case | HR (95% CI^a^) | Case | HR (95% CI^a^) | Case | HR (95% CI^a^) |
| Age 20 and 50 |  |  |  |  |  |  |  |  |  |  |  |  |  |  |  |  |
| < 18.5 | 8 | 1.56 (0.77-3.15) | 13 | 1.14 (0.64-2.02) |  | 13 | 1.49 (0.84-2.64) | 9 | 1.18 (0.61-2.30) |  | 2 | － | 15 | 1.24 (0.73-2.12) | 2 | － |
| ≥ 18.5-25 | 563 | Reference | 558 | Reference |  | 769 | Reference | 528 | Reference |  | 129 | Reference | 830 | Reference | 249 | Reference |
| ≥ 25-30 | 282 | 1.04 (0.89-1.21) | 239 | 1.23 (1.05-1.44) |  | 346 | 1.25 (1.09-1.44) | 248 | 1.18 (1.00-1.38) |  | 63 | 0.78 (0.57-1.08) | 374 | 1.39 (1.22-1.59) | 115 | 1.09 (0.86-1.40) |
| ≥ 30 | 37 | 1.41 (0.99-2.00) | 65 | 1.50 (1.15-1.96) |  | 63 | 1.31 (1.00-1.71) | 45 | 1.57 (1.14-2.16) |  | 13 | 1.69 (0.92-3.12) | 67 | 1.71 (1.32-2.23) | 19 | 0.85 (0.52-1.39) |
| *P*_trend_ |  | 0.249 |  | < 0.001 |  |  | 0.002 |  | < 0.001 |  |  | 0.928 |  | < 0.001 |  | 0.947 |
| Continuous, per 5 kg m^-2^ |  | 1.08 (0.95-1.22) |  | 1.21 (1.08-1.35) |  |  | 1.17 (1.06-1.30) |  | 1.20 (1.06-1.35) |  |  | 0.99 (0.77-1.27) |  | 1.33 (1.20-1.46) |  | 1.01 (0.84-1.20) |
| Age 20 and baseline |  |  |  |  |  |  |  |  |  |  |  |  |  |  |  |  |
| < 18.5 | 5 | － | 6 | 1.19 (0.49-2.90) |  | 9 | 1.47 (0.73-2.98) | 4 | － |  | 1 | － | 9 | 1.55 (0.77-3.13) | 2 | － |
| ≥ 18.5-25 | 501 | Reference | 490 | Reference |  | 682 | Reference | 447 | Reference |  | 112 | Reference | 725 | Reference | 208 | Reference |
| ≥ 25-30 | 330 | 1.07 (0.93-1.25) | 296 | 1.04 (0.89-1.20) |  | 409 | 1.21 (1.06-1.37) | 320 | 1.10 (0.95-1.28) |  | 72 | 0.89 (0.65-1.23) | 464 | 1.21 (1.07-1.37) | 151 | 1.14 (0.92-1.43) |
| ≥ 30 | 54 | 1.20 (0.89-1.61) | 83 | 1.30 (1.03-1.65) |  | 91 | 1.13 (0.90-1.41) | 59 | 1.52 (1.15-2.01) |  | 22 | 0.81 (0.50-1.32) | 88 | 1.60 (1.27-2.02) | 24 | 1.00 (0.65-1.53) |
| *P*_trend_ |  | 0.206 |  | 0.085 |  |  | 0.027 |  | 0.012 |  |  | 0.374 |  | < 0.001 |  | 0.547 |
| Continuous, per 5 kg m^-2^ |  | 1.08 (0.96-1.21) |  | 1.10 (0.99-1.22) |  |  | 1.11 (1.01-1.21) |  | 1.16 (1.03-1.30) |  |  | 0.91 (0.74-1.12) |  | 1.23 (1.12-1.35) |  | 1.05 (0.89-1.24) |
| Age 50 and baseline |  |  |  |  |  |  |  |  |  |  |  |  |  |  |  |  |
| < 18.5 | 5 | － | 6 | 1.03 (0.42-2.52) |  | 8 | 1.31 (0.65-2.65) | 4 | － |  | 1 | － | 8 | 1.21 (0.57-2.58) | 1 | － |
| ≥ 18.5-25 | 293 | Reference | 309 | Reference |  | 406 | Reference | 276 | Reference |  | 61 | Reference | 443 | Reference | 135 | Reference |
| ≥ 25-30 | 429 | 1.01 (0.87-1.18) | 379 | 1.05 (0.90-1.23) |  | 548 | 1.07 (0.93-1.22) | 392 | 1.08 (0.92-1.27) |  | 94 | 1.10 (0.78-1.54) | 592 | 1.10 (0.97-1.25) | 188 | 1.12 (0.88-1.41) |
| ≥ 30 | 163 | 1.11 (0.91-1.36) | 181 | 1.37 (1.13-1.65) |  | 229 | 1.24 (1.05-1.47) | 158 | 1.46 (1.19-1.79) |  | 51 | 0.96 (0.65-1.42) | 243 | 1.45 (1.23-1.71) | 61 | 1.32 (0.96-1.81) |
| *P*_trend_ |  | 0.487 |  | 0.004 |  |  | 0.025 |  | 0.002 |  |  | 0.901 |  | < 0.001 |  | 0.094 |
| Continuous, per 5 kg m^-2^ |  | 1.04 (0.94-1.15) |  | 1.15 (1.05-1.26) |  |  | 1.10 (1.01-1.19) |  | 1.18 (1.06-1.31) |  |  | 0.99 (0.82-1.19) |  | 1.18 (1.09-1.28) |  | 1.14 (0.98-1.33) |

Abbreviations: HR, hazard ratio; CI, confidence intervals.

Left-side CRC: rectum, sigmoid colon, descending colon and splenic flexure of colon.

Right-side CRC: transverse colon, ascending colon and cecum.

Early stage: stage I/II.
Advanced stage: stage III/IV.
^a^ Adjusted for randomization arm (intervention or control), sex (male or female), study center, race (white, black, Hispanic or Asian), family history of CRC (yes or no) and cigarette smoking status (yes or no).

Table S10. Association of demographic characteristics and BMI trajectories in relation to CRC incidence in the PLCO study

| BMI trajectory | Randomization arm | | | |  | Sex | | | |  | Family history of CRC | | | |  | Cigarette smoking status | | | |
| --- | --- | --- | --- | --- | --- | --- | --- | --- | --- | --- | --- | --- | --- | --- | --- | --- | --- | --- | --- |
|  | Intervention (N = 901) | | Control (N = 1,130) | |  | Male (N = 1,196) | | Female (N = 835) | |  | No (N = 1,676) | | Yes (N = 256) | |  | No (N = 834) | | Yes (N = 1,197) | |
|  | Case | HR (95% CI^a^) | Case | HR (95% CI^a^) |  | Case | HR (95% CI^a^) | Case | HR (95% CI^a^) |  | Case | HR (95% CI^a^) | Case | HR (95% CI^a^) |  | Case | HR (95% CI^a^) | Case | HR (95% CI^a^) |
| Normal BMI | 276 | Reference | 332 | Reference |  | 271 | Reference | 337 | Reference |  | 508 | Reference | 71 | Reference |  | 251 | Reference | 357 | Reference |
| Normal BMI to overweight | 433 | 1.05 (0.90-1.23) | 575 | 1.15 (1.00-1.33) |  | 655 | 1.05 (0.91-1.22) | 353 | 1.19 (1.02-1.39) |  | 826 | 1.08 (0.96-1.21) | 134 | 1.30 (0.97-1.74) |  | 421 | 1.25 (1.06-1.47) | 587 | 1.01 (0.88-1.16) |
| Normal BMI to obese | 158 | 1.19 (0.97-1.46) | 187 | 1.17 (0.97-1.41) |  | 235 | 1.19 (1.00-1.42) | 110 | 1.14 (0.91-1.42) |  | 290 | 1.19 (1.02-1.37) | 37 | 1.13 (0.75-1.69) |  | 137 | 1.26 (1.08-1.57) | 208 | 1.12 (0.94-1.34) |
| overweight BMI to obese | 34 | 1.35 (0.93-1.95) | 36 | 1.21 (0.85-1.73) |  | 35 | 1.32 (0.92-1.91) | 35 | 1.25 (0.87-1.77) |  | 52 | 1.14 (0.86-1.52) | 14 | 2.25 (1.27-4.00) |  | 25 | 1.02 (0.71-1.68) | 45 | 1.39 (1.01-1.91) |
| *P*_trend_ |  | 0.058 |  | 0.039 |  |  | 0.031 |  | 0.047 |  |  | 0.028 |  | 0.045 |  |  | 0.043 |  | 0.043 |

Abbreviations: HR, hazard ratio; CI, confidence intervals.

^a^ Adjusted for randomization arm (intervention or control), sex (male or female), study center, race (white, black, Hispanic or Asian), family history of CRC (yes or no) and cigarette smoking status (yes or no).

Table S11. Association of CRC characteristics and BMI trajectories in relation to CRC incidence in the PLCO study

| BMI trajectory | Location | | | |  | Stage | | | |  | Grade | | | | | |
| --- | --- | --- | --- | --- | --- | --- | --- | --- | --- | --- | --- | --- | --- | --- | --- | --- |
|  | Left-sided (N = 890) | | Right-sided (N = 875) | |  | Early stage (N = 1,191) | | Advanced stage(N = 830) | |  | Well (N = 207) | | Moderate (N = 1,286) | | Poor (N = 385) | |
|  | Case | HR (95% CI^a^) | Case | HR (95% CI^a^) |  | Case | HR (95% CI^a^) | Case | HR (95% CI^a^) |  | Case | HR (95% CI^a^) | Case | HR (95% CI^a^) | Case | HR (95% CI^a^) |
| Normal BMI | 261 | Reference | 273 | Reference |  | 365 | Reference | 240 | Reference |  | 56 | Reference | 387 | Reference | 123 | Reference |
| Normal BMI to overweight | 456 | 0.98 (0.84-1.15) | 409 | 1.11 (0.95-1.31) |  | 584 | 1.02 (0.89-1.18) | 421 | 1.12 (0.95-1.32) |  | 101 | 1.17 (0.86-1.66) | 640 | 1.07 (0.94-1.22) | 193 | 1.10 (0.86-1.41) |
| Normal BMI to obese | 145 | 0.94 (0.76-1.12) | 157 | 1.01 (0.82-1.24) |  | 201 | 0.93 (0.77-1.11) | 142 | 1.21 (0.97-1.52) |  | 40 | 0.68 (0.44-1.04) | 223 | 1.08 (0.91-1.28) | 55 | 1.29 (0.91-1.82) |
| overweight BMI to obese | 28 | 0.77 (0.51-1.15) | 36 | 0.99 (0.69-1.41) |  | 41 | 1.04 (0.74-1.46) | 27 | 0.97 (0.65-1.46) |  | 10 | 0.90 (0.45-1.78) | 36 | 1.34 (0.93-1.92) | 14 | 0.82 (0.46-1.46) |
| *P*_trend_ |  | 0.267 |  | 0.969 |  |  | 0.621 |  | 0.234 |  |  | 0.130 |  | 0.157 |  | 0.614 |

Abbreviations: HR, hazard ratio; CI, confidence intervals.

Left-side CRC: rectum, sigmoid colon, descending colon and splenic flexure of colon.

Right-side CRC: transverse colon, ascending colon and cecum.

Early stage: stage I/II.
Advanced stage: stage III/IV.
^a^ Adjusted for randomization arm (intervention or control), sex (male or female), study center, race (white, black, Hispanic or Asian), family history of CRC (yes or no) and cigarette smoking status (yes or no).
